# Effect of Melatonin as an Antioxidant Drug to Reverse Hepatic Steatosis: Experimental Model

**DOI:** 10.1155/2020/7315253

**Published:** 2020-06-03

**Authors:** Blanca Martínez Soriano, Antonio Güemes, Guillermo Pola, Azucena Gonzalo, Pilar Palacios Gasós, Ana C. Navarro, Roberto Martínez-Beamonte, Jesús Osada, José J. García

**Affiliations:** ^1^Department of Surgery, University Hospital Lozano Blesa, Zaragoza, Spain; ^2^Department of Surgery, General Defense Hospital, Zaragoza, Spain; ^3^CIBER (Biomedical Net Investigation Centre), Obesity and Nutrition Physiopathology, Madrid, Spain; ^4^Department of Biochemistry and Molecular and Cellular Biology, University of Zaragoza, Zaragoza, Spain; ^5^Department of Physiology, University of Zaragoza, Zaragoza, Spain

## Abstract

**Introduction:**

The hepatic steatosis of the nonalcoholic origin or NAFLD is increasing at present, particularly in Western countries, parallel to the increase in obesity, constituting one of the most prevalent hepatic processes in the Western society. Melatonin has been successfully tested in experimental models in mice as a drug capable of reversing steatosis. The effect of melatonin on fat metabolism can be summarized as a decrease in lipid peroxidation and a decrease in oxidative stress, biochemical phenomena intimately related to fat deposition in the hepatocyte. There are hardly any studies in large animals.

**Objective:**

In this study, we investigate the effects of melatonin administered orally at a dose of 10 mg/kg/day to reverse established hepatic steatosis induced by a special diet in a porcine animal model.

**Materials and Methods:**

We analyze the parameters of oxidative stress: malondialdehyde (MDA), 4-hydroxyalkenals (4-HDA), and carbonyls, degree of fat infiltration (analyzed by direct vision by a pathologist and by means of a computer program of image treatment), and serological parameters of lipid metabolism and hepatic damage. These parameters were analyzed in animals to which hepatic steatosis was induced by means of dietary modifications.

**Results:**

We have not been able to demonstrate globally a beneficial effect of melatonin in the improvement or reversal of liver steatosis once established, induced by diet in a porcine animal model. However, we have found several signs of improvement at the histological level, at the level of lipid metabolism, and at the level of oxidative stress parameters. We have verified in our study that, in the histological analysis of the liver sample by means of the program image treatment (free of subjectivity) of the animals that continue with the diet, those that consume melatonin do not increase steatosis as much as those that do not consume it significantly (*p*=0.002). Regarding the parameters of oxidative stress, MDA modifies in a significant manner within the group of animals that continue with the diet and take melatonin (*p*=0.004). As for lipid metabolism, animals that maintain the steatotic diet and take melatonin lower total and LDL cholesterol levels and increase HDL levels, although these results do not acquire statistical significance.

**Conclusions:**

In this study, it has not been possible to demonstrate a beneficial effect of melatonin in the improvement or reversal of liver steatosis once established and induced by diet in the porcine model. It is true that signs of improvement have been found at the histological level, at the level of lipid metabolism, and at the level of oxidative stress phenomena, when comparing animals with established steatosis that are treated with melatonin with those who do not take it. This work is the first study conducted in a large animal model in which the effect of melatonin is studied as a treatment in the reversal of established hepatic steatosis.

## 1. Introduction

Nonalcoholic fatty liver disease (NAFLD) is characterized by the accumulation of fatty acids, triglycerides, and cholesterol in the cytoplasm of the hepatocyte. It occurs in subjects who do not drink alcohol or drink moderately (<20 g/day) and is considered as the expression in the liver of a complex syndrome called “metabolic syndrome.” NAFLD includes two clinical entities: nonalcoholic fatty liver (NAFL), which refers to the presence of hepatic steatosis without evidence of hepatocellular damage or fibrosis, and nonalcoholic steatohepatitis (NASH), which is the presence of hepatic steatosis that is associated with inflammation and liver damage with or without fibrosis. NASH may progress to cirrhosis, liver failure, and hepatocarcinoma [1].

The prevalence of NAFLD is not well known and is probably underestimated. This is because most patients remain asymptomatic or have discrete biological alterations, the absence of precise serological markers, and the need for liver biopsy for definitive diagnosis. However, we know that steatosis is one of the most prevalent liver diseases in the Western world, linked to the increase in obesity and metabolic syndrome, and varies widely depending on the population studied. Two Japanese studies [2, 3] published an incidence in the general population of 31 and 86 cases, respectively, of suspected NAFLD per 1000 persons/year. In severely obese patients undergoing bariatric surgery, the prevalence of NAFLD may exceed 90% and up to 5% may have cirrhosis.

The treatment of hepatic steatosis once established is achieved primarily through dietary restrictions and lifestyle changes [4–7]. Drugs used in the treatment of metabolic syndrome (antidiabetics, statins, etc.) have also been used [8]. Recently, the efficacy of various antioxidant agents, such as vitamin E and melatonin [9–11], has been demonstrated in the treatment of hepatic steatosis. There is currently no specific harmless and effective treatment for hepatic steatosis.

Melatonin is a natural hormone synthesized by the pineal gland in animals. Melatonin synthesis is not found exclusively in the pineal gland, as its secretion has been described in numerous peripheral organs such as the retina, bone marrow, skin, and gastrointestinal tract and in some cells such as lymphocytes and platelets [12, 13].

One of the main properties of melatonin is its powerful antioxidant effect [12], which is attributed to its ability to neutralize free radicals and its indirect detoxifying action by stimulating antioxidant enzymes [14]. It has been proven that melatonin supplements could protect against some diseases such as atherosclerosis, cancer, and Alzheimer's disease. At the level of hepatic metabolism, melatonin has been tested as a drug to prevent the process of ischemia-reperfusion [15] and also as a treatment of liver damage by toxins such as alcohol, carbon tetrachloride, aflatoxin, or chemotherapy agents [16–19].

Melatonin has been shown to be beneficial in the treatment and prevention of NAFLD in murine experimental models. Experimental studies have been carried out in rodents, the majority with the intention of preventing diet-induced hepatic steatosis, observing a decrease in blood lipids, an improvement in hepatic enzymes, and a decrease in oxidative stress parameters, as well as an improvement in histology [20–25]. There are hardly any studies in large or small animals that prove this effect. In humans, studies have been carried out that demonstrate the lipid lowering and cytoprotective capacity of melatonin in hepatic steatosis once established, although it is true that there are hardly any studies that demonstrate histological improvement [26–29].

Our working hypothesis is based on the fact that melatonin is an effective treatment to reverse established and induced hepatic steatosis by means of a special diet in a porcine animal model, as well as in the murine model. The aim of our study is to assess the effect of melatonin administered orally on lipid metabolism, hepatic histology, and oxidative stress parameters.

## 2. Material and Methods

### 2.1. Model and Sample: An Experimental Animal

We use the great English white pig as an experimental animal. This model was chosen as such because it is consistent with the hepatic physiology of the human. The animals were obtained from a breeding farm for animal experimentation, which were free of diseases or parasites and with an approximate weight of 45 kg and an approximate age of 6 months. All animals that did not meet a minimum weight (30 kg) or that showed signs of diseases (diarrhoea, adynamia, dermatosis, and abnormal behaviour) were excluded from the experiment. The animals were kept in cages, shared by 3 or 4 animals, and provided with feeders and water. The study was approved by the Advisory Ethics Commission for Animal Experimentation of the University of Zaragoza.

### 2.2. Experimental Design and Study Groups

The induction of steatosis was carried out by means of a special diet, without using any pharmacological agent. Its main characteristics were as follows: high content in saturated fat (25%), deficiency in methionine and choline, and supplemented with 2% cholesterol plus 0.5% sodium cholate. The animals belonging to study groups 1 and 3 received melatonin doses of 10 mg/kg/day orally for 4 weeks. The drug was acquired in powder form, which was encapsulated in a sucrose excipient. It was administered in a single dose by means of capsules that were ingested at the same time as the diet described above, mixing the drug with part of the diet in such a way as to ensure complete intake.

A control group consisted of 6 animals; healthy animals without steatosis underwent a normal nonsteatosis diet and underwent a single surgical intervention for the purpose of obtaining samples, whose values served as a reference. 31 animals were part of the study. For 3 months, they were on an original diet of our group specially designed to produce steatosis, at the end of which, they underwent an open biopsy (to obtain sufficient samples). After the surgical intervention, the animals were assigned to 4 study groups: group 1 (*N* = 12): animals that maintained a steatotic diet for 1 more month and received concomitant treatment during this month with melatonin (10 mg/kg/day); group 2 (*N* = 9): animals that maintained a steatotic diet for one more month and received no drug; group 3 (*N* = 5): animals that did not continue with the steatotic diet but received melatonin treatment for one month at the same dose; group 4 (*N* = 5): animals that did not continue with the steatotic diet and did not receive any drug. After this time, the animals underwent a second surgical intervention to obtain the same samples of liver tissue, blood, and serum. After this second operation, the animals were slaughtered.

In each surgical intervention, a minilaparotomy was performed to observe the macroscopic characteristics of the liver and to take a sample of hepatic tissue from segment IV. This sample was divided into two parts: one of them was kept in formaldehyde for anatomopathological study. Another sample was frozen and preserved in liquid nitrogen to obtain oxidative stress parameters: MDA and carbonyls. During the intervention, a sample of 20 ml of venous blood extracted from the portal vein or the vena cava was also obtained. The blood was centrifuged at 3000 rpm for 3 min to obtain serum. Biochemical parameters were obtained from the blood sample.

### 2.3. Induction of Hepatic Steatosis

In the experimental porcine model, hepatic steatosis can be induced by means of a specially developed and proven diet that resembles the induction of steatosis in humans. This is without the need to use drugs or toxic products. The induction of steatosis was carried out through a special diet whose main characteristics were as follows:

- High saturated fat content (25%)
- Deficient in methionine (<1/4 of the needs) and choline (1/6 of the needs)
- Supplemented with 2% cholesterol and 0.5% sodium cholate.The following table shows its composition

### 2.4. Histological Analysis of Liver Biopsy

To determine the degree of hepatic steatosis, we used two methods. Method 1 is a conventional semiquantitative analysis in which the pathologist estimates the percentage of steatosis under direct vision (HEX ×1000) of each sample expressed in %. Method 2 is a method of quantification via computer-assisted analysis consisting of an estimate of the area of computer-assisted steatosis of samples obtained and previously selected.

The program measures the degree of steatosis by analyzing tonalities from photographs taken of each sample of hepatic tissue, without areas occupied by vessels and other structures. We use a specific software assisted by Matlab®, created by the research group itself to perform a digital analysis of the biopsy samples. It has three fundamental functions:

- Cal-estea.m: it is the main function that performs the calculation of steatosis
- Filter-m: it is the function of the gray filter, which allows the program to be adapted to the wide range of shades that can be presented to us in the sample
- Region-m: it is the function that eliminates regions that do not correspond to fat (for example, veins with white tones)

### 2.5. Measurement of Oxidative Stress Parameters: MDA and Carbonyls

The concentration of MDA + 4-hydroxyalkenals (4-HDA) was determined by a colourimetric method based on the reaction of a chromogenic reagent, N-methyl-2-phenylindole, with MDA or with 4-HDA, at a temperature of 45°C. The condensation of one MDA or 4-HDA molecule with two molecules of N-methyl-2-phenylindole produces a stable chromophore which, in the presence of methanesulfonic acid, has a maximum absorbance at 586 nm [30].

In order to evaluate the oxidative damage to the proteins of the homogenized ones, the determination of the carbonyl remains of the proteins was used. This method is based on the reaction of the carbonyl remains of proteins with dinitrophenylhydrazine (DNPH), forming a derivative that is quantified by measuring its absorbance in the range 360–390 nm. During the procedure, trichloroacetic acid (TCA) is used for the precipitation of proteins, washes for the removal of excess DNPH that has not reacted with carbonyl residues, and guanidine to redissolve the proteins in order to facilitate spectrophotometer readings.

From the absorbance obtained, the concentration of carbonyl moieties was calculated using the Beer–Lambert law and the molar absorption coefficient of DNPH (*Ɛ* = 22,000 M-1 × cm^−1^). Finally, after the determination of the total proteins, the results were expressed in nmol of carbonyl moieties/mg of total proteins.

The laboratory equipment included a spectrophotometer with disposable plastic cuvettes to measure the visible range and quartz cuvettes to measure in the ultraviolet (UV) range.

### 2.6. Measurement of Biochemical Parameters

The measurement of serological parameters of hepatic function (aspartate aminotransferase (AST), alanine aminotransferase (ALT), gamma-glutamyl transferase (GGT), alkaline phosphatase (FA), and bilirubin (B)) and plasma lipid concentration (total cholesterol, HDL cholesterol, and LDL cholesterol) was performed by automatic processors for automatic reading of concentrations of liver lipids and enzymes.

### 2.7. Variables to Be Studied

The following variables were studied: the weight of the animal, steatosis reached, blood lipids (triglycerides (TG), total cholesterol, HDL cholesterol, and LDL cholesterol), hepatic enzymes (aspartate aminotransferase (AST), alanine aminotransferase (ALT), gamma-glutamyl transferase (GGT), alkaline-FA phosphatase, and bilirubin), parameters of oxidative stress (MDA and carbonyls), and histology (degree of fat infiltration).

### 2.8. Statistic Analysis

To analyze the relationship between the variables of a study, a bivariate analysis is carried out. To study the linear relationship between two quantitative variables, the Pearson or Spearman correlation coefficient is used. The correlation coefficient can take values between −1 and 1, indicating the zero correlation between the variables under study. To evaluate the differences between the first biopsy or pre and the second biopsy or post, in relation to the quantitative variables of the study, means comparison methods are used for related samples. Wilcoxon is used when the variable does not follow the normal distribution, and Student's *t*-test is used when there is normality.

To quantify the difference between both time periods, we calculate the percentage of change. Positive results will indicate increased values in the postbiopsy and negative results will indicate decreased values.

## 3. Results

### 3.1. Steatosis Reached

The total of 31 (100%) animals in the study group presented significant hepatic steatosis in the first biopsy, moderate (steatosis 30–59%) and severe (steatosis >60%) after being on a special diet for 3 months, both in the measurement made by the pathologist and in the measurement made by the computer program.

**Figure 1:**
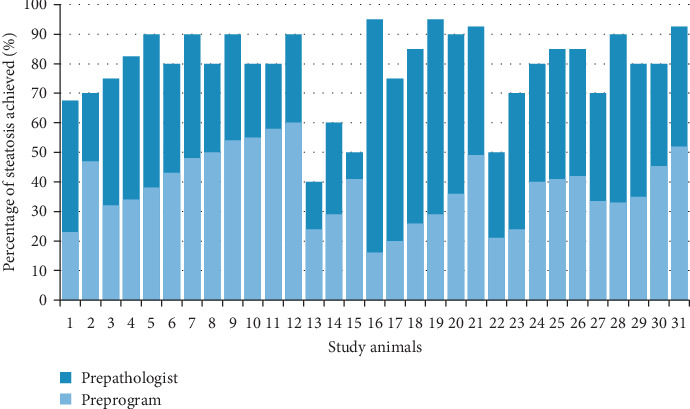
Average steatosis reached by each of the animals, according to the pathologist and program.

Figure 1 shows the average degree of steatosis of the animals for each of the animals according to pathologist analysis and digital image analysis. There are no statistically significant differences in the average steatosis reached per group.

It is observed that the degree of steatosis according to the digital analysis provides in all cases average values lower than those provided according to an analysis by a pathologist. On average, the degree of steatosis according to a pathologist scores 40.68 (DE = 14.66) more than that according to the digital analysis at the premoment.

**Figure 2:**
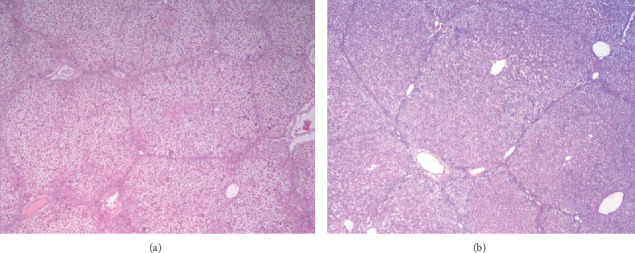
Optical microscopy image, HE 2x dyeing with 95% steatosis (a) and optical microscopy image, HE 2x dyeing with 40% steatosis (b).

Figure 2 shows the optical microscopy image of the animal with the highest percentage of steatosis being seen by a pathologist (95% of steatosis) and the optical microscopy image of the animal with the lowest degree of steatosis being seen under direct vision by a pathologist (40% of steatosis).

### 3.2. Weight

**Figure 3:**
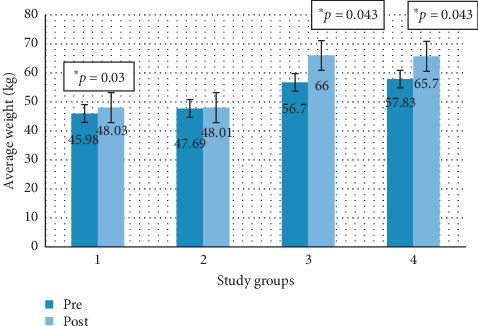
Average pre-post weight (kg) by study groups. Group 1: animals that maintained a steatotic diet and received concomitant treatment. Group 2: animals that maintained a steatotic diet and received no drug. Group 3: animals that did not continue with the steatotic diet but received melatonin treatment. Group 4: animals that did not continue with the steatotic diet and did not receive any drug.

In all groups, there was an increase in average weight between the two time periods evaluated, as shown in Figure 3, this increase being statistically significant in groups 1, 3, and 4 (*p*=0.03, *p*=0.043, and *p*=0.043, respectively).

In all groups, the percentage change (% difference in weight at the end of the study with respect to the initial weight) was studied, calculated as follows:

(1) $$\begin{array}{c} \%\text{ weight change}=\frac{\text{postweight}-\text{preweight}}{\text{preweight}}. \end{array}$$

### 3.3. Biochemical Variables: Triglycerides, Total Cholesterol, HDL, LDL, AST, ALT, GGT, and FA

**Figure 4:**
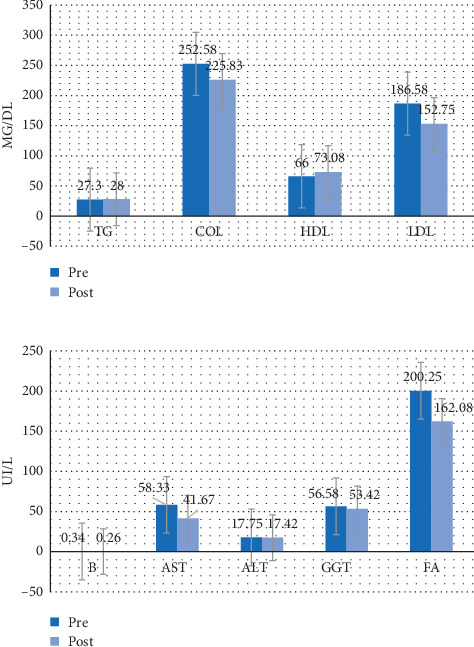
Average values of the pre-post parameters for group 1. TG = triglycerides, COL = total cholesterol, HDL = HDL cholesterol, LDL = LDL cholesterol, B = bilirubin, AST = aspartate aminotransferase, ALT = alanine aminotransferase, GGT = gamma-glutamyl transferase, FA = alkaline phosphatase.

**Figure 5:**
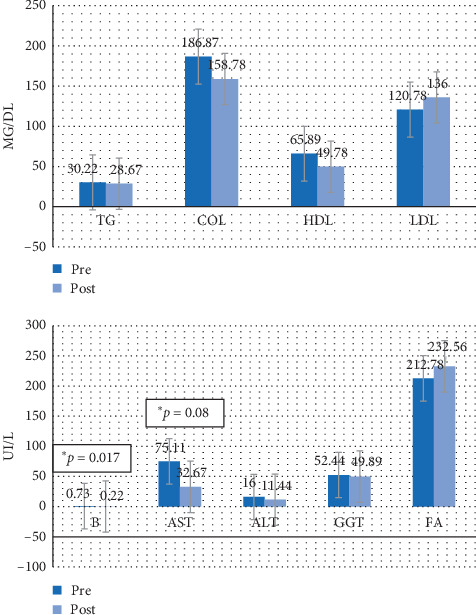
Average values of the pre-post parameters for group 2. TG = triglycerides, COL = total cholesterol, HDL = HDL cholesterol, LDL = LDL cholesterol, B = bilirubin, AST = aspartate aminotransferase, ALT = alanine aminotransferase, GGT = gamma-glutamyl transferase, FA = alkaline phosphatase.

**Figure 6:**
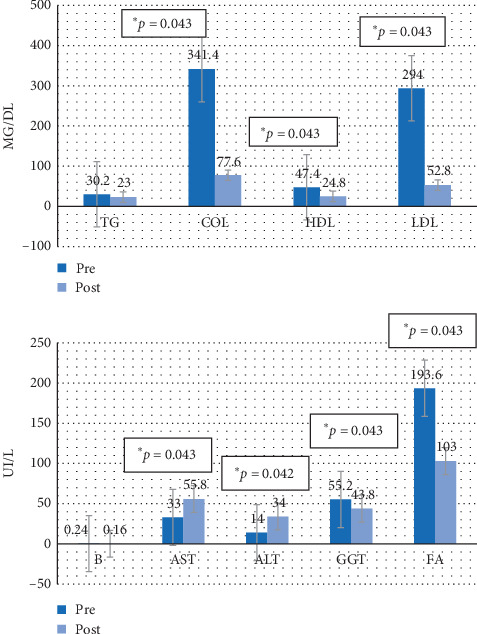
Average values of the pre-post parameters for group 3. TG = triglycerides, COL = total cholesterol, HDL = HDL cholesterol, LDL = LDL cholesterol, B = bilirubin, AST = aspartate aminotransferase, ALT = alanine aminotransferase, GGT = gamma-glutamyl transferase, FA = alkaline phosphatase.

**Figure 7:**
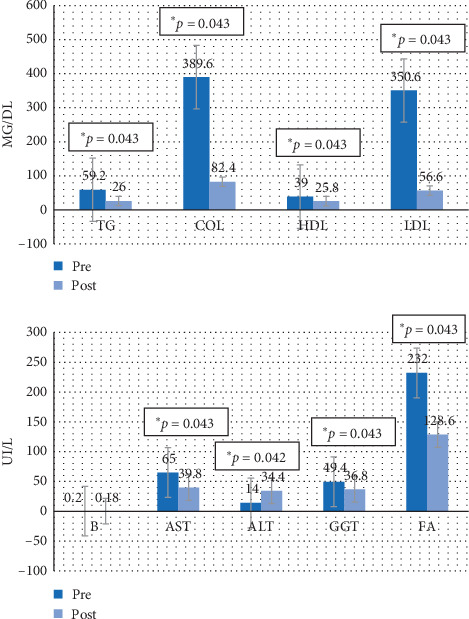
Average values of the pre-post parameters for group 4. TG = triglycerides, COL = total cholesterol, HDL = HDL cholesterol, LDL = LDL cholesterol, B = bilirubin, AST = aspartate aminotransferase, ALT = alanine aminotransferase, GGT = gamma-glutamyl transferase, FA = alkaline phosphatase.

Figures 4–7 show the mean prevalue and postvalue of each of the parameters evaluated for each of the study groups.

#### 3.3.1. Group 1

In group 1 (the group that continued with the steatotic diet and was treated with melatonin), no parameter shows statistically significant differences (*p* > 0.05) before and after treatment.

#### 3.3.2. Group 2

In group 2 (the group that continued with the steatotic diet and was not treated with melatonin), the average AST decreased significantly (*p*=0.008), going from 75.11 UI/L (DE = 47.29) to 32.67 UI/L (DE = 13.93); there is also a significant decrease (*p*=0.017) of the average B going from 0.73 UI/L (DE = 0.83) to 0.22 (DE = 0.26).

#### 3.3.3. Group 3

In group 3 (the group that discontinued the diet and was treated with melatonin), the average values of total cholesterol, HDL, LDL, GGT, and FA decreased at the end of the study period in a statistically significant way, compared with the values at the beginning (*p*=0.043 in all cases). However, we observe a significant increase in ALT and AST (*p*=0.043 and *p*=0.042 respectively).

#### 3.3.4. Group 4

In group 4 (the group that discontinued the steatotic diet and was not treated with melatonin), the average values of TG, total cholesterol, HDL, LDL, AST, GGT, and FA also decreased at the end of the study period, presenting significant differences between the two study time periods (pre and post) (*p*=0.043 in all cases). However there is a significant increase in ALT (*p*=0.042).

### 3.4. Analysis of Hepatic Fat Infiltration

**Figure 8:**
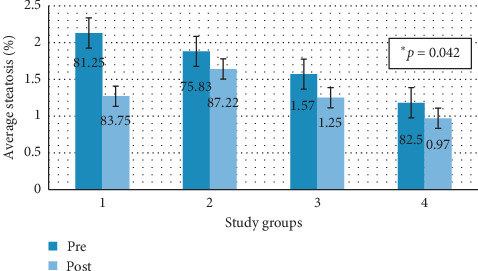
Average pre-post steatosis by study groups. Group 1: animals that maintained a steatotic diet and received concomitant treatment. Group 2: animals that maintained a steatotic diet and received no drug. Group 3: animals that did not continue with the steatotic diet but received melatonin treatment. Group 4: animals that did not continue with the steatotic diet and did not receive any drug.

Figure 8 shows graphically the degree of average steatosis by groups in the two study time periods analyzed by the direct vision of the pathologist.

A decrease in steatosis was observed in groups 3 and 4, and an increase in steatosis in groups 1 and 2, with a significant decrease in group 4 (*p*=0.042), the animals that discontinued their diet and were not treated with melatonin.

**Figure 9:**
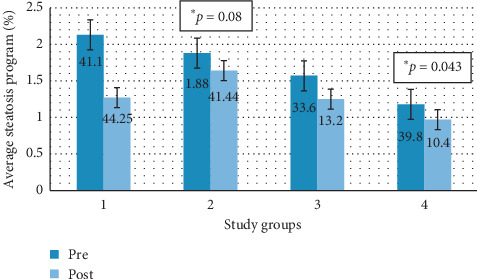
Percentage of average pre-post steatosis (program) by study groups. Group 1: animals that maintained a steatotic diet and received concomitant treatment. Group 2: animals that maintained a steatotic diet and received no drug. Group 3: animals that did not continue with the steatotic diet but received melatonin treatment. Group 4: animals that did not continue with the steatotic diet and did not receive any drug.

Figure 9 shows graphically the degree of average steatosis by groups in the two study time periods digitally analyzed by the image processing software.

There is a decrease in steatosis in groups 3 and 4, and there is an increase in steatosis in groups 1 and 2. This increase in group 2 is statistically significant (*p*=0.08), as is the decrease in group 4 (*p*=0.043).

**Figure 10:**
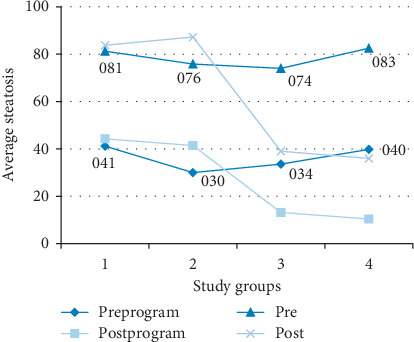
Percentage of average pre-post steatosis (program) by study groups. Group 1: animals that maintained a steatotic diet and received concomitant treatment. Group 2: animals that maintained a steatotic diet and received no drug. Group 3: animals that did not continue with the steatotic diet but received melatonin treatment. Group 4: animals that did not continue with the steatotic diet and did not receive any drug.

The degree of steatosis measured by the program provides lower average values in relation to the degree of steatosis measured by the pathologist. On average, the degree of steatosis measured by the direct vision of the pathologist [31] scores 40.68% more in the premoment and 36.87% more in the postmoment. In Figure 10, it is possible to observe, in a graphical way, the average values of the degree of steatosis of both measurements by groups for both periods of study (pre and post) and how the same tendency is observed for the two methods of measurement employed.

**Figure 11:**
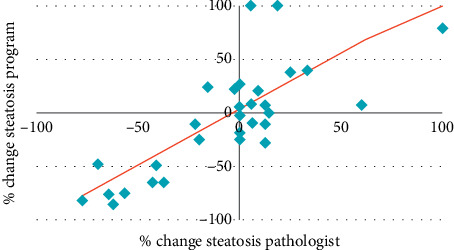
Scatter diagram. % change steatosis program−% change steatosis pathologist.

Figure 11 shows the relationship between the percentage of change obtained with the program and the percentage of change obtained with the pathologist.

The percentage of change of steatosis between the pretime and posttime of the follow-up of the subjects shows a strong linear relationship between both methods, obtaining a correlation coefficient of 0.767 with a level of significance being lower than 0.001.

### 3.5. MDA

**Figure 12:**
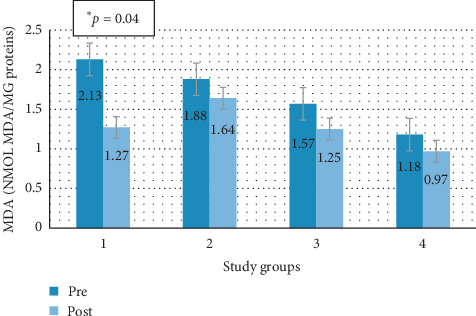
Average pre-post MDA by study groups. Group 1: animals that maintained a steatotic diet and received concomitant treatment. Group 2: animals that maintained a steatotic diet and received no drug. Group 3: animals that did not continue with the steatotic diet but received melatonin treatment. Group 4: animals that did not continue with the steatotic diet and did not receive any drug.

Figure 12 shows graphically the degree of average MDA by groups in the two study periods.

In all study groups, there was a decrease in the average value of MDA, being statistically significant in group 1 (*p*=0.04), the subjects that continued the steatotic diet and were given melatonin.

### 3.6. Carbonyls

**Figure 13:**
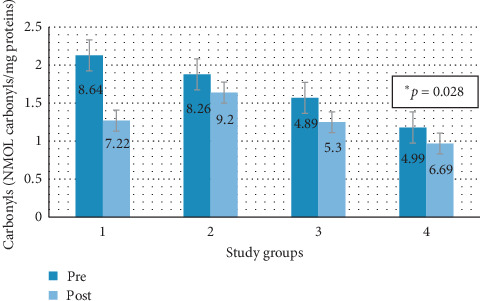
Medium pre-post carbonyls by study groups. Group 1: animals that maintained a steatotic diet and received concomitant treatment. Group 2: animals that maintained a steatotic diet and received no drug. Group 3: animals that did not continue with the steatotic diet but received melatonin treatment. Group 4: animals that did not continue with the steatotic diet and did not receive any drug.

Figure 13 shows graphically the average carbonyl degree by groups in the two study periods.

In groups 2, 3, and 4, there is an average increase of carbonyls, being only significant in group 4 (*p*=0.028). The small average decrease in group 1 (subjects that continue on a diet and are treated with melatonin) does not have statistical significance, as in the case of MDA.

## 4. Discussion

NAFLD is one of the most prevalent hepatic processes in the Western society and one of the major causes of liver failure in the Western world. Usually associated with, but not exclusively to, obesity and the metabolic syndrome, it could lead to a real epidemic in the future. Numerous authors have shown that the reduction of excess weight and the consequent resistance to insulin can restore liver physiology and histology [8,9]. But at the present time, there is no effective pharmacological treatment of NAFLD that is not linked to a lifestyle change, including weight loss. Many drugs, antioxidants, hepatoprotectors, vitamin complexes, free radical scavengers, etc. have been tested with the idea of either avoiding the accumulation of lipids in the hepatocyte or extracting lipids from cells, showing beneficial effects.

Although more studies are needed to define their usefulness in NAFLD [32–37], the discovery of a drug with these properties could change the spectrum and impact of the disease.

Melatonin, the universal hormone that is produced in different concentrations, in almost all our anatomy, has been studied as one of the possible solutions to the alteration of lipid metabolism and fat deposition in the hepatocyte [12]. Experimental studies have shown an action of melatonin on fat metabolism, which can be summarized as a decrease in lipid peroxidation and a decrease in oxidative stress [13], biochemical phenomena intimately related to the accumulation of lipids in the hepatocyte. Other effects that have been attributed to melatonin are as follows: stimulation of antioxidant enzymes in hepatocytes, regulation of antioxidant enzyme transcription genes, scavenging of oxygen free radicals, stimulation of glutathione synthesis, increase in the activity of other antioxidant molecules, decrease in the generation of free radicals in mitochondria, decrease in the expression of proteins that have an effect on the accumulation of lipids in the hepatocyte cytoplasm, protection of cell membranes against lipid peroxidation, and reduction of proinflammatory factors responsible for the progression of NAFLD to NASH.

Several experimental models have been described to resemble human NAFLD. The ideal model would be one that reflects both the histology and the physiopathology of the disease in its different stages. Of course, this model should be reproducible, reliable, simple, predictable, and economical. Most of these models have been described in rodents. There are few models of hepatic steatosis in large animals, and in most of them, liver damage has been induced by the administration of toxins such as alcohol [38,39].

In 2009, Lee et al. described, for the first time, a NASH model in large animals produced by dietary manipulation using Ossabaw miniature pigs [40]. This model is not very useful due to the restriction of the use of these animals. In our work, we used an experimental model, the pig White-Landrace, in which we reproduced steatosis. This model is original and sufficiently proven in previous studies of our group and that has the particularity that only by means of dietetic manipulation, without using drugs or toxics, controlled and very high degrees of steatosis are achieved. We have not found any model published thus far that induces, without the use of substances harmful to the liver or an aggressive diet (excess of polyunsaturated, fructose fats, or the deficit of elements necessary for beta-oxidation such as choline and methionine), a degree of macrovesicular steatosis similar to ours, in such a short time and with minimal repercussions on the physiology of the animal. Another strength of our experimental model is that it allows us to check liver damage and quantify steatosis by performing liver biopsies before and after the development of steatosis; in many murine models, this is not possible, and in humans, no author performs them.

All steatogenic diets described induce an increase in the weight of the animal. In our study, all animals increased in weight after the introduction of the steatogenic diet. The data is consistent with the data found in the literature (Ossabaw pigs). The effects of melatonin were null on the weight of the animal.

In order to explore the effect on the lipid metabolism of melatonin, we studied lipid peroxidation (LPO) on the polyunsaturated fatty acids of the hepatocyte (during the process of accumulation of lipids in the hepatocyte, the action of free radicals on the lipids takes place mainly on the polyunsaturated fatty acids of cellular samples, causing their peroxidation). The final products of this process of LPO are aldehydes, hydrocarbon gases, and various chemical residues, with MDA and 4-HDA being the majority. Therefore, the concentration of MDA+4-HDA is an indicator of the degree of peroxidation of the lipids of the biological membranes [41].

In our study, we found that both MDA and carbonyls decreased in the groups that were administered melatonin with respect to those who did not receive it; therefore, this indicates an effect of the hormone on lipid metabolism at the intracellular level.

Plasma levels of cholesterol and triglycerides increased in animals subjected to steatogenic diet but were not modified by the effect of melatonin with statistical significance. However, we can observe that animals that continue with the diet and take melatonin (group 1) exhibit lowered LDL and cholesterol levels and increased HDL levels. This could indicate some protective effect of melatonin in this group of animals, although it lacks statistical significance. In the literature, we find works that ratify the lipid-lowering effect of melatonin [22, 24, 28, 42, 43]. One reason that might argue this result is that, in our experimental model, we start from established hepatic steatosis, with chronically elevated blood lipid levels, a situation that does not occur in the other experimental studies, since melatonin is administered concomitantly with the administration of the high-fat diet as prevention.

With respect to the levels of liver enzymes (ALT and GGT), bilirubin, and FA, in our study, their levels have not been altered by the action of melatonin. In other experimental studies on murine models, Pan et al. [20] were able to demonstrate that the administration of intraperitoneal melatonin at doses of 5 to 10 mg/kg/day for 12 days was effective in reducing serum ALT and AST. The difference with our work is that the previous authors gave jointly the steatotic diet and melatonin to the experimental animals and our work starts from animals with an already established NAFLD; that is to say, perhaps melatonin could not have a preventive effect of the hepatic lesion when it is administered early (before the accumulation of fat in the hepatocyte), being its effect null when NAFLD is already established.

The work carried out by Hatzis et al. [21] gives us some light in this respect. The authors were able to demonstrate that hepatic cell necrosis was significantly lower in rats that had received a diet rich in fats and melatonin at doses of 5 mg or 10 mg/kg/day via intraperitoneal for 4–8 weeks, finding that the levels of AST and ALT were lower after receiving the drug. However, in their work, a group of animals underwent induced steatosis (through diet) before being treated with melatonin, because this last group of animals did not obtain any hepatic cytoprotective effect, like the animals in our study. It can be concluded that melatonin is ineffective as a treatment once NAFLD is established. In our work, the degrees of NAFLD reached after the diet were very high, much more than those obtained in similar studies with other experimental models; perhaps with more moderate degrees of fat infiltration, melatonin could have some effect, and this extreme should be investigated.

The traditional method of evaluation of the degree of hepatic steatosis, used by most authors in the world, is performed by evaluating the histological sample stained with Hematoxylin-Eosin evaluation performed more or less subjectively by a pathologist. To avoid subjectivity and improve accuracy, we also use a method of evaluation by digital image analysis through a program created by the team itself, to contrast this subjectivity. In our study, it was observed that the measurement of steatosis by digital analysis provides in all cases some average values lower than those obtained by the direct vision of the pathologist. Studies published in the literature corroborate this overestimation [44]. An explanation of this overestimation, as several authors explain, is that the measured area of hepatocyte vacuoles is better measured by computer methods than the purely visual way, since the computer is capable of eliminating areas of the sample with a similar visual aspect (in the design of our computer program, we took this fact into account). In our study, the percentage change of steatosis between the pretime and posttime of subject tracking shows a strong linear relationship between the two methods. In conclusion, in our study, we obtained a high correlation between both methods, but with an overestimation of the degree of steatosis by the pathologist analysis.

In our study, melatonin was unable to restore histology, not even to the low steatosis figures at baseline, although we observed the phenomenon that the degree of fat infiltration, which increased gradually if the animals continued to take the steatotic diet, remained stable if they were given melatonin, with significant differences. These data corroborate experimental data in murine models. Hatzis et al. [21] demonstrated a protective effect of melatonin when administered synchronously with a high-fat diet; however, this effect could not be demonstrated in the group of rats that received melatonin at a late stage of the experiment, i.e., as a treatment once hepatic steatosis was established. Pan et al. [20] also showed that a moderate or high dose of melatonin (5–10 mg/kg/day) improved the degree of hepatic steatosis when administered concomitantly to the high-fat diet.

Human studies have the handicap that they do not perform biopsies to corroborate the improvement of hepatic steatosis after the administration of melatonin, probably because of the not inconsiderable percentage of complications that this diagnostic method entails.

With this work, we can demonstrate a protective effect of melatonin in terms of the lower progression of steatosis in the histological sample; however, we cannot show improvement, as in other studies. We also discovered a hypolipidemic effect of melatonin, since animals that maintain the steatotic diet and take melatonin reduce their levels of total and LDL cholesterol whilst increasing their levels of HDL cholesterol, although these results are not statistically significant. As previously stated, in our work, we start from very high levels of steatosis. Rodents in the experimental studies were administered melatonin concomitantly with the fat-rich diet, so these studies aim to find a protective effect of melatonin in the establishment of hepatic steatosis, not the reversal of hepatic steatosis, which is the aim of our study. On the other hand, in studies carried out in humans, the degree of steatosis from which we start does not reach the very high levels of steatosis from which we start. It is, therefore, possible that melatonin has a preventive effect on liver injury or even serves as a treatment for hepatic steatosis when the degree of fat involvement of hepatocytes is lower, being difficult to reverse when the hepatocyte involvement is the majority and does not cease the stimulus that produced it.

Another aspect to keep in mind is the melatonin administration time. We have seen that in our study positive results are obtained at the molecular level, in the intimate mechanism of production of NAFLD; that is to say, we observe that melatonin attenuates oxidative stress and lipid peroxidation in groups of animals that continue with the diet; however, the changes at a histological level have only been demonstrated at the time of preventing progression. This could be because the period of administration of melatonin (4 weeks) or the dose administered (10 mg/kg/day) is insufficient to observe changes in the accumulation of hepatocyte lipids at the histological level. Studies with a longer administration time of melatonin or with a higher dose would be needed to demonstrate this effect.

We believe that one of the greatest strengths of our study was the experimental model used. It is original from our group who are experienced in another work [45]. In addition, we tried to individualize the effect of melatonin from the effect of the intake of the steatotic diet. Many of the papers do not include groups with discontinuation of the diet or seek purely the protective effect of the drug administered in conjunction with the diet. In our work, we preferred to use 4 different groups of animals, which forced us to use a considerable number of experimental animals but allowed us to simulate the different scenarios in which melatonin can be administered as a drug to treat NAFLD.

## Data Availability

The data used to support this study are provided in the Supplementary Materials. The complementary data can be obtained from the corresponding author upon request.
